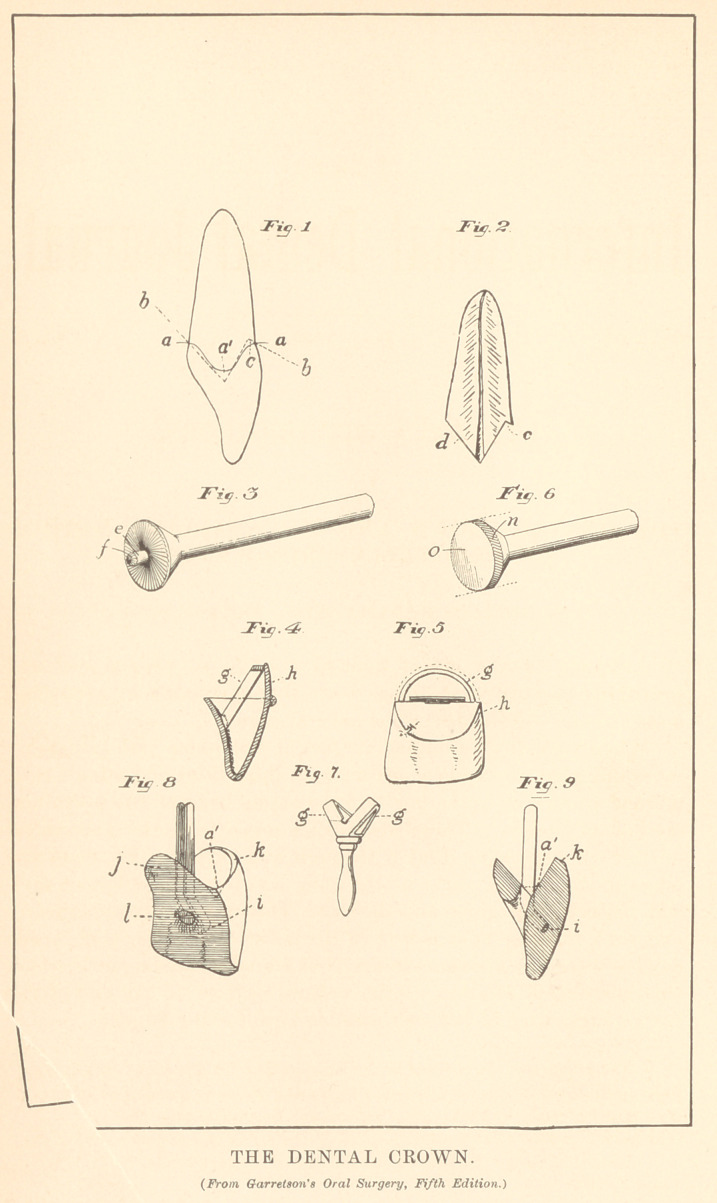# The Dental Crown and Its Enabling Method of Removable Bridge-Work

**Published:** 1890-05

**Authors:** William H. Gates

**Affiliations:** Philadelphia


### International Dental Journal.

Vol. XI. May, 1890. No. 5.

### Original Communications.[1]

### THE DENTAL CROWN AND ITS ENABLING METHOD OF REMOVABLE BRIDGE-WORK.[2]

BY WILLIAM H. GATES, D.D.S., PHILADELPHIA.

Advance in the art of dentistry, while so pronounced and gratifying in most respects, has been singularly slow in producing a satisfactory substitute for the natural crown.

When carefully examined, in fact, it will seem not a little remarkable that the radical errors and absurdities of that old-time method known as "pivoting" are the actual basis of the prevailing method of to-day. These absurdities arose from the anatomical mistake of supposing that a through and through cut, from the labial to the lingual curve of the gum, was the proper line of division between the crown and the root. This mistake was manifestly excusable coming at a period of dental science when such crude devices were its exponents in art; but we must no longer overlook this anatomical relation, as the natural outline of the end of the root is suggestive of the best possible form for the crown.

---

[1] The editor and publishers are not responsible for the views of authors of papers published in this department, nor for any claim to novelty, or otherwise, that may be made by them. No papers will be received for this department that have appeared in any other journal published in this country.

[2] Read before the Odontological Society of Pennsylvania, Saturday, February 1, 1890.

The distinctive features of pivoting were the concave foundation in the end of the root and the tooth rudely joined therein by the dowel, or "pivot," as it came to be called from its tendency to turn; but these are likewise the basal features of the so-called porcelain crowns. In both—the Gates-Bonwill and the Logan—the change relates only to the *make-up* of the so-called crown and its pivot, the outer part being of porcelain, while the basal portion of both alike is of plastic material added thereto because of its adaptability to the pivot joint in the end of the root. To have termed either of them a *crown* was incorrect, as no crown has a convex base. They are simply composite pivot-teeth, and the post of each is the core of its pivot.

An objection to the existing pivoting method is, that it cuts away the natural base susceptible of being clasped like a rock and interposes in its place a base that throws the entire support upon the post-pivot, and thereby forms an adverse leverage within the end of the root, the extreme length of the composite pivot-tooth being the long arm, and the radius of the end of the root the short arm of the lever. This tendency to split the root becomes still greater through the vertical diameter, as the tooth, confined by the post between the lateral inclined planes, acts as a positive wedge. In view of these facts, which are witnessed also by the immense strength required in the platinum posts, we must certainly have increased respect for the stanch qualities of root material that has so rarely given way under such adverse conditions.

A further objection to this pivoting method arises from the impossibility of making a proper joint where there is no guide but guesswork; no opportunity to see or know the relation of the two surfaces when applied; no guide for the proper occlusion with the opposite jaw; no recourse, indeed, but to cut and try for some sort of makeshift.

Because of these difficulties the suggestion of the amalgam base was thought to be a great boon, and the porcelain was countersunk to receive it. There was a grievous disappointment, however, because of the mechanical impossibility of getting the amalgam to a proper position in so large a single quantity, which soon became apparent. Resort was then had to the perishable plastics, because less rigid before setting, a gold band being employed to protect them, and likewise act as a *bandage* for the ever-threatened root.

But why must we infringe on the pericementum, establish bacteria nests, and disfigure the tooth with this bandage of gold?

If we take nature's suggestion we will replace the natural crown with a substitute that presents a true mechanical joint, into which the end of the root projects, and which is therefore properly called a crown. Such a crown I have provided, and hence have termed it *the dental crown.*

Referring to the drawings for its illustration (see plate), the line *a a*, Fig. 1, shows the anatomical outline and the strong defensive angle of the end of the root, while the dotted line *b b* indicates the slight modification necessary to make this angle available as a perfect crown-seat, a ledge, *c*, Figs. 1 and 2, being formed at the outer base against which the crown may solidly abut. This ledge neutralizes the inclined plane which would imperil the crown, and as the crown seat represents a trifle less than a right angle, the plane *d* supplements in the most ample and positive manner the support afforded by the ledge, with the result that we have here a mechanical crown-seat of the very highest order, affording permanent protection both to the root and to the crown.

To make this crown-seat, a facing-wheel, Fig. 3, and a gauge, Figs. 4 and 5, are provided, which are very exact and practical in application, because each has a supplemental form that allows one plane to be finished before the other is begun. The facing-wheel is file-cut on its front face. Its diameter covers one face of the crown-seat, which it quickly and definitely forms while firmly sustained by a self-centring point, *e*, Fig. 3, which, separately made, has been hermetically sealed into its mandrel; and as this centring point cuts only on its front face, *f*, Fig. 3, which is also its largest diameter, the wheel is thereby confined to the selected position while free to adjust the plane of the crown-seat to any inclination desired.

The gauge, made of sheet metal, represents a kind of skeleton of the body and the outer lobe only of the crown, the body of the lobe being represented by a circular crib, *g*, Figs. 4 and 5, over which its outer face, *h*, rests as a hinged cover. The natural crown having been reduced to the line of the gum, and the enamel removed, this gauge is simply applied against the end and labial face of the root while forming the outer face of the crown-seat by the facing-wheel. The occlusion with the opposite jaw, the mouth being closed, determines at once the length of the crown required; and by uncovering the crib in testing, to be sure of its proper contact, this outer and determining face of the crown-seat is carefully formed, the contour of the gauge proving the position correct. Thus we have an unfailing guide, a direct open view, and a thoroughly-controlled, painless, and effective instrument for overcoming, right at the start, the paramount and, by the old method, most formidable difficulties of crown-setting,—viz., obtaining the correct position and the proper adaptation.

The supplemental or face-extension wheel, Fig. 6, is of similar diameter to the facing-wheel, but cuts only on its peripheral face. Resting flat on the plane already formed, it simply extends that face of the crown-seat as far up under the free edge of the gum as the selected crown requires. Of this form there may be a pair, cutting right and left.

The supplemental gauge, Fig. 7, is an oval band or crib bent edgewise to the standard angle. Guided by the outer face, this quickly determines the correct position of the inner face of the crown-seat, which is formed by a facing-wheel in the right angle attachment.

It will be observed that the coronal end of the crown-seat extends beyond the enamel line at the ridge. This, like that at the ledge, is a useful variation from the saddle-shaped, anatomical outline of the root, as it projects a solid line of support of unexampled height into the centre of the crown. It is slightly trimmed at each end to admit its reception between the lateral wings *a′* of the crown so provided.

The crown itself requires no trimming. It represents the standard of joint as suited to the crown-seat. The special advantage, in an economic as well as an artistic sense, in having at hand perfectly-adjusted crowns in porcelain, need not be enlarged upon. It may be mentioned here that in the manufacture of this crown it is contemplated to embed into the porcelain just within the peripheral border of the angle a slight rib, or a perforated mat of platinum, in order always to have exact uniformity of the gauge.

An important feature also embodied in this crown consists in employing a staple as the bond between the crown and the root. Entering the inner face of the crown, its loop is bent backward and embedded in the porcelain towards the outer face as shown at *i*, Fig. 8, thus securing the most powerful combination with the crown-seat. This arrangement provides an open passage directly through the crown and the pulp-canal while the crown is in place upon the root, and affords facilities for the attainment of highly-important purposes.

Instead of having the approach to the canal sealed with impenetrable porcelain, it is a manifest advantage to have an opportunity to reopen in case of pathological conditions not apparent at the time of closing; and the open passage is of still greater importance at the time of setting the crown, for it permits the centre of the crown to remain open and undisturbed until the treatment can be made under favorable circumstances. Thus, instead of wasting time at the start in a difficult diagnosis of the case, the crown-seat is at once formed, the canal enlarged, and the crown set with gutta-percha; the presence of a slender pin embedded there leaves the canal open when withdrawn; and on removing the crown at a subsequent sitting, the treatment is made with every advantage of free access, an open view, and the opportunity to make make deliberately whatever tests may be necessary.

This open passage also makes available an important discovery touching the material employed in the setting. Gutta-percha, having shown unusual tenacity and power of resistance as a thin layer between this crown and its crown-seat, requires only the assistance of a firmly-embedded staple to make it serve as a permanent setting. But this direct access is essential in order to embed the staple properly.

The convenience and simplicity of such an adjustment is instantly apparent. A thin layer of gutta-percha, adapted to the base of the crown, is warmed and impressed upon the crown-seat, which, being moist, allows the crown to withdraw the gutta-percha as an impression. The excess at the border and immediately around the staple being trimmed away, it is warmed and replaced until the crown practically rests against the crown-seat. At this point, if the mounting is to be temporary, two slender sticks of gutta-percha are warmed and pressed within and upon the opposite edges of the staple, and embedding also a small ordinary pin between them. The point of this pin, made blunt, will be slightly in advance of the gutta-percha when inserted into the canal, and the gutta-percha should be at least an eighth of an inch in advance of the ends of the staple, so that in condensing it may obtain firm contact with the sides and retaining-points of the canal. The canal and root having been well dried, the loosening and withdrawal of the pin will leave the case in a safe and successful condition. But the permanent placing is still more simple, as, when the gutta-purcha setting has been adjusted to the base of the crown and the root dried, nothing remains but to partly fill the canal with a soft mix of amalgam, carry the crown to place after warming, and finish by thoroughly introducing very dry amalgam until mercury will no longer appear.

In making this permanent closing of the canal the insertion and withdrawal of a slender steel pin, reaching almost to the foramen, will leave a closed tube in the amalgam for more convenient approach in case of necessity.

It should here be stated that amalgam itself is perfectly adapted for setting this crown, as a soft mix, placed between the crown and crown-seat, permits the crown to go fully and easily to place, leaving only a mere film of amalgam between, the excess of mercury being withdrawn at the same time with that in the canal; and the only part of the joint facing outward, namely, the ledge, is easily provided with a fender excluding the amalgam, so as to appear, if at all, only as a fine line of gold. But the extraordinary convenience, entire concealment, and stanch character of gutta-percha as a cement between two solid surfaces, otherwise sustained in close apposition, make amalgam unnecessary, and add to the laurels of this invaluable material, gutta-percha.

The reaming of the canal is preferably deferred until the crown-seat is formed, as any improved position desirable to be given to the crown will require ordinarily only a slight corresponding change in the position of the staple at the mouth of the canal without resort to bending; and since the dislodging force against the crown, hitherto born by the post, is here sustained by the crown-seat with its immense reserve of force, we can widen laterally, or make such other shaping of the pulp-canal as suits any purpose of advanced ideas.

But the question will be asked, How shall the position of the crown-seat be determined when the end of the root is far decayed or already cut into concave shape? Simply by inserting into the pulp-canal, temporarily, a pivot of orange wood, the outer end of which, cut to a wedge-shape, will give support to the centring bar of the facing-wheel. In such cases, after the crown, set for a few days upon these partial outlines with a slight excess of gutta-percha, has pressed the morbid gum back to its normal condition, a section of corrugated tubing, having its outer end likewise cut to wedge-shape, is to be permanently set into the enlarged canal by means of amalgam, which is then simply built out far enough to restore the crown-seat. As all tendency to split the root is interrupted by this simple arrangement, it is obvious that roots otherwise impossible of preservation may in this manner be saved. Boxes of these wedge-pointed pivots and sections of corrugated tubing may be kept at hand.

Lastly, the facility of the open canal offers a unique advantage for bridge-work. All banding is set aside and a strong, simple, and removable attachment is made to this crown. A lingual gold face set into the crown its own thickness, *j*, Fig. 8, including a short hood at the cutting end, is easily secured by a suitable screw, *l*, Fig. 8, provided with a platinum counterpart previously embedded in amalgam in the pulp-canal.

In conclusion, it gives me pleasure to say that I have this method of mounting bridge-work protected in the United States Patent Office for the profession, and that they shall not be subject to the payment of office rights for the use thereof.

## Figures and Tables

**Figure f1:**